# Prolonged Vitality of Small-pox Contagion

**Published:** 1856-10

**Authors:** S. E. McKinley

**Affiliations:** Crook’s Cross Roads, Hancock Co., Ga.


### *Prolonged Vitality of Small-pox Contagion.* By S. E. McKinley.

To the Editor of the Examiner :—

Dear Sir,—It is sometimes instructive, and at all times interesting to the *literati* in medicine, to read of remarkable eccentricities of things pertaining to medical science, whether they have a direct bearing upon *practical medicine*, the great *ultimatum* of the science, or upon the intricate and diversified mazes of its theory. Whatever is commonplace, however momentous, is beheld with indifference, and scarcely merits, in the sovereign opinion of the multitude, a passing remark. On the contrary, whatever assumes the character of novelty, anything making a departure from the ordinary course of operations, is beheld with astonishment.

The terrors of yellow fever in one section of our country, are scarcely felt by its "fixed inhabitants;" yet yellow fever is none the less destructive because it is a household word there; nor is its cause any better known by reason of its frequent visitation; yet every person, almost, pretends to cure it. In some measure the same remark is applicable to cholera; but more especially is it so to that loathsome disease named after the presumptuous shepherd of king Alcithons—Syphilis. This demon has its centre everywhere, nor do the extremest bounds mark with any certainty the limits of a remotely probable circumference. It is ubiquitous; its habitat is everywhere; it descends from the lofty mansion to the dweller in the lowly cot, and in return radiates from the purlieus of vice, midst ribald congregations of abandoned wretches, to the polished inmate of the lordly palace. With all our familiarity with these "very popular" diseases, what do we know of them? We have been taught their pathology, arrest and cure; their eccentricities, peculiarities, and caprices we know; we can go no farther. Reason has its legitimate province; a doctrine may be above and beyond, but not opposed to our reason.

Permit me to offer a familiar illustration of our defective, if not our positive want of knowledge of the "*virus variolæ habitudo.*" It goes to establish, beyond the remotest doubt, that atmosphere impregnated with the ethero-volatile principle of small-pox, may be confined in and hover about certain other things, and finally, after the lapse of years, induce the very disease from which it first arose.

In the year 1851, small-pox prevailed somewhat extensively in Oglethorpe Co., in this State; it was not confined to villages alone, but introduced itself upon the more rural inhabitants of the inner country, and disturbed the otherwise prevailing quiet. During this year, a family attacked by it, had, after their recovery, their house cleansed and white-washed, and all clothes worn by the sick either burned or destroyed. Notwithstanding this, it re-appeared in the same family last July, 1856.

Three years after *the first* visitation, the wife of the husband died, and quite recently he married again. The new wife, upon being installed empress of the household *de facto et de jure*, was inspired with similar sentiments to those of the redoubtable Crockett when first elected to Congress, viz.: to "bring about a radical change in the judiciary." True to her ideas of reform, and to bring the household under the auspices of a different system of diplomacy, she instituted and brought into immediate action a host of legislative operations, which led to the most astounding developments. Bed clothes, coats, trowsers, (old and new,) vests, shirts, drawers, stockings, hats, bonnets, shawls, dresses, etc., carpets, desks, bureaus, cupboards, and various other articles were disturbed in their quiet security, and exposed to the solar elements and soap and water. Very shortly after this tearing up of things, this universal exhumation, and when just about to enjoy the full fruition of the reform system, this amiable and good woman was taken with confluent small-pox, together with a servant, who did not have it in 1851. Neither wife or servant had been from home, nor had any suspicious person been to see them. They had not been to or passed through any town. They lived five miles out in the country from Lexington, and had no intercourse whatever with any person from whom they could have taken it. The writer has been requested to notice this in some journal, and if it be of any importance it is at your service.

*Crook's Cross Roads, Hancock Co., Ga.*

